# Protein-ligand binding affinity prediction using multi-instance learning with docking structures

**DOI:** 10.3389/fphar.2024.1518875

**Published:** 2025-01-03

**Authors:** Hyojin Kim, Heesung Shim, Aditya Ranganath, Stewart He, Garrett Stevenson, Jonathan E. Allen

**Affiliations:** ^1^ Center for Applied Scientific Computing, Lawrence Livermore National Laboratory, Livermore, CA, United States; ^2^ Biosciences and Biotechnology Division, Lawrence Livermore National Laboratory, Livermore, CA, United States; ^3^ Global Security Computing Applications Division, Lawrence Livermore National Laboratory, Livermore, CA, United States; ^4^ Computational Engineering Division, Lawrence Livermore National Laboratory, Livermore, CA, United States

**Keywords:** AI-driven drug development, virtual high-throughput screening, protein-ligand interaction, molecular docking, 3D atomic graph representation, structure-based machine learning, multi-instance learning, attention mechanism

## Abstract

**Introduction:**

Recent advances in 3D structure-based deep learning approaches demonstrate improved accuracy in predicting protein-ligand binding affinity in drug discovery. These methods complement physics-based computational modeling such as molecular docking for virtual high-throughput screening. Despite recent advances and improved predictive performance, most methods in this category primarily rely on utilizing co-crystal complex structures and experimentally measured binding affinities as both input and output data for model training. Nevertheless, co-crystal complex structures are not readily available and the inaccurate predicted structures from molecular docking can degrade the accuracy of the machine learning methods.

**Methods:**

We introduce a novel structure-based inference method utilizing multiple molecular docking poses for each complex entity. Our proposed method employs multi-instance learning with an attention network to predict binding affinity from a collection of docking poses.

**Results:**

We validate our method using multiple datasets, including PDBbind and compounds targeting the main protease of SARS-CoV-2. The results demonstrate that our method leveraging docking poses is competitive with other state-of-the-art inference models that depend on co-crystal structures.

**Discussion:**

This method offers binding affinity prediction without requiring co-crystal structures, thereby increasing its applicability to protein targets lacking such data.

## 1 Introduction

Given the vast number of drug-like chemical compounds and the rapid growth of compound library databases, it is crucial to develop an effective tool for large-scale virtual high-throughput screening to identify hit molecules. To address this need, recent studies have employed various machine learning and deep learning-based inference models to enhance traditional computational modeling techniques such as molecular docking and molecular dynamics simulations for accurately predicting protein-ligand interactions and binding affinities.

One major application utilizing machine learning techniques in drug discovery involves the use of SMILES strings and their associated features such as molecular descriptors (RDKit, 2024; Moriwaki et al., 2018) and fingerprints (Rogers and Hahn, 2010), to predict compound properties and target-specific interactions. This includes predicting toxicity, solubility, membrane permeability, binding affinity, and other compound characteristics associated with the target receptors. The approaches in this category use a variety of machine learning methods, ranging from random forest algorithms to various neural network architectures (Minnich et al., 2020; Ramsundar et al., 2019).

Recent studies use advanced architectures and techniques with atomic graph representations such as graph neural networks (Duvenaud et al., 2015; Stärk et al., 2022a), graph transformers and attention mechanisms (Rong et al., 2020) in unsupervised or self-supervised settings. These approaches show the advantages of utilizing pretrained models on unlabeled large-volume compound databases. Furthermore, large language model-based approaches have been proposed to address pretrainng and finetuning with the unlabeled compound data (Chithrananda et al., 2020; Mazuz et al., 2023).

Another direction in leveraging deep learning techniques uses 3D atomic representations of the protein-ligand structures to predict the interaction and binding affinities. These structure-based methods use 3D convolutional neural networks (3DCNNs) utilizing voxelized atomic representations of co-crystal complex structures to predict binding affinities (Gomes et al., 2017; Ragoza et al., 2017; Jiménez et al., 2018; Zhang et al., 2019) and to predict protein-binding sites (Jiménez et al., 2017). Furthermore, graph neural networks (GNNs), graph convolutional networks (GCNs), and spatial graph convolutional neural networks (SGCNNs) have been introduced for binding affinity prediction. These models utilize graph representations of atomic data from co-crystal complex structures, capturing atoms and their connectivity through nodes and edges (Feinberg et al., 2018; 2019; Zhang et al., 2023). Recently, equivariant attention networks have received attention for representing 3D point cloud or graph data under rotations such as SE(3)-transformers (Fuchs et al., 2020). Satorras et al. developed light-weight E(n) equivariant graph neural networks (EGNNs) equivariant to rotations, translations, reflections and permutations without the need for higher-order representations (Satorras et al., 2021). Powers et al. proposed to use data-efficient E(3) equivariant networks with point cloud data to guide how to attach new molecular fragments in molecule optimization (Powers et al., 2022). Scantlebury et al. proposed a lightweight E(n)-equivariant graph neural network for machine learning-based scoring functions (Scantlebury et al., 2023).

An additional recent trend involves using hybrid neural network architectures or fusion networks to capture diverse input representations and interactions. Jones et al. proposed a fusion method to complement 3DCNN and SGCNN architectures to effectively capture both spatial information and atom connectivities (Jones et al., 2021). Jiang et al. proposed the use of two independent graph network modules to capture intra- and intermolecular interactions (Jiang et al., 2021). Kyro et al. proposed the use of hybrid attentions with 3DCNN and GCN (Kyro et al., 2023). Mqawass et al. proposed a fusion method of different input representations (Mqawass and Popov, 2024).

Most methods in this category rely on co-crystal complex structures of protein-ligand data to train the neural network models. However, crystal structures are not readily available in the early stages of most drug discovery applications. The PDBbind dataset (Su et al., 2019), a meticulously curated subset of the Protein Data Bank (PDB) (Burley et al., 2019) containing experimental binding affinity data, is virtually the sole dataset accessible for structure-based protein-ligand learning tasks. For that reason, we often utilize simulated docking poses of protein-ligand complex structures for structure-based deep learning inference models. The docking poses can be generated using molecular docking tools such as AutoDock VINA (Trott and Olson, 2010; Eberhardt et al., 2021), and GLIDE (Friesner et al., 2004; Halgren et al., 2004). Recent studies have proposed the use of deep neural network approaches to serve as docking scoring functions (McNutt et al., 2021), or to generate docking poses including DeepDock (Méndez-Lucio et al., 2021), EquiBind (Stärk et al., 2022b), TankBind (Lu et al., 2022), DiffDock (Corso et al., 2023), and UniMol (Zhou et al., 2023).

While generating docking poses is feasible and practical, training or evaluating deep neural network models on these poses results in inaccurate predictions, as the docking poses are considered “noisy” data. To identify inaccurate docking poses, Shim et al. proposed a pose classification approach using two different neural network methods, 3DCNN and point cloud network (PCN) (Shim et al., 2022). While the method filters out inaccurate docking poses; it requires an additional computational or inference method for binding affinity estimation. Another study developed a pose classification method without using crystal structures to improve the correlation between docking scores and experimental data (Shim et al., 2024). However, their method relies on the docking poses and their scoring functions. A recently proposed method leverages an E(3)-invariant graph neural network, combined with docking and binding affinity data, to address binding affinity prediction for kinase compounds (Backenköhler et al., 2024). Although this approach utilizes template docking to achieve more accurate poses, it does not explicitly address the distinction between accurate and inaccurate docking data, particularly in scenarios where a significant amount of inaccurate docking data is present.

In this paper, we present a novel inference method based on multi-instance learning (MIL) that utilizes a set of docking poses for each protein-ligand entity to predict binding affinity. Multi-instance learning is a type of weakly supervised learning used to predict a label for a group (bag) of instances, where the individual labels of the instances within the bag are not available (Carbonneau et al., 2018). In the standard MIL setup for classification, it is assumed that negative bags exclusively contain negative instances, while positive bags contain at least one positive instance. This technique offers flexibility by mitigating the potential problems associated with ambiguous and time-consuming labeling, compared to the traditional supervised learning. For more detail about MIL methods in various computer vision and machine learning applications, refer to the following review papers (Carbonneau et al., 2018; Fatima et al., 2023).

We leverage MIL-based regression to predict binding affinities when only molecular docking data is available, as there are no accessible co-crystal structures. Our method begins with graph neural networks, specifically SGCNN and EGNN, representing each ligand pose alongside its associated target receptor instance as a graph embedding. We then extract latent features from multiple pose instances per complex entity using these graph networks. Finally, we integrate these features into our pose-wise attention network, which functions as a MIL framework to predict binding affinities for each complex entity. We train and evaluate our models on molecular docking structures that may contain inaccurate poses. We demonstrate our method using the PDBbind and SARS-CoV-2 main protease datasets. The primary contribution of our method is as follows:

• Our method does not rely on co-crystal structures, which increases applicability across various drug target applications.

• Multiple docking poses per complex structure entity can be utilized together, without the need to filter out inaccurate ones.

• Our pose-wise attention mechanism, which can be integrated with various backbone network architectures, enhances binding affinity prediction even with docking pose data only, compared to existing scoring methods including top-pose-based and non-weighted average scores.

## 2 Methods

### 2.1 Problem definition

Most high-throughput screening and 3D structure-based neural network approaches utilize co-crystal complex structures or the “best” poses with the highest docking scores (lowest binding energy). Instead, we consider all generated poses with the target receptor as a bag for each complex structure entity. We formulate this problem as multi-instance learning (MIL) regression to predict a single real-value label for a bag (Dooly et al., 2002). Each bag represents a single protein-ligand structure entity consisting of multiple docking poses, $X=\left\{x_{1},\ldots ,x_{K}\right\}$ , with at least one instance being close to the target label y . It is assumed that the sequence of docking pose instances can be random. The real-value labels represent the binding affinities. Each instance $x_{i}\in R^{D}$ represents the atomic representation of the docking pose with the protein receptor, including the 3D coordinates of the atoms and their atomic features. The number of docking poses K can vary depending on the molecular docking methods, generally ranging from 10 to 20 in our applications. Our goal is to predict the binding affinity y by leveraging our proposed MIL network framework M together with the backbone graph network G to interpret a set of docking pose and the receptor instances X .

### 2.2 Input data and featurization

For input ligand docking poses and protein receptors, we extract atomic features presented by Pafnucy (Stepniewska-Dziubinska et al., 2018). This atomic feature set was widely used in various structure-based machine learning methods (Jones et al., 2021; Kyro et al., 2023; Shim et al., 2022; 2024). Each atom feature consists of 3D coordinates (x,y,z) of the atom position and its features comprising a 19 dimension feature vector, which includes element type, atom hybridization, number of heavy atom bonds, bond properties (hydrophobic, aromatic, acceptor, donor, ring), partial charge, and Van der Waals radii. All atomic coordinates within each protein-ligand structure are normalized, aligning them with the center of the ligand. We used Open Babel (O’Boyle et al., 2011) for format conversion and partial charge calculation, and utilized the tfbio package (tfbio, 2018) to extract protein, ligand, and binding co-complex features.

### 2.3 Model architecture

We utilize graph neural network architectures capable of leveraging graph representations of protein-ligand structures to capture both atomic features and their interconnections, which serves as a backbone model. The backbone networks provide low-dimensional K embedding representation $H=\left\{h_{1},\ldots ,h_{K}\right\}$ for a bag of multiple docking pose instances. Thus, $h_{k}=G_{\psi }\left(x_{k}\right)$ , where $G_{\psi }\left(\cdot \right)$ is a graph neural network with parameters ψ . The low-dimensional embedding H is used as input features in the MIL-based attention network for final prediction.

For the backbone graph neural networks, we use atomic-level graph representations, where nodes represent protein or ligand atoms, and edges are connectivity (bonds) between these atoms. Each node encapsulates its corresponding atomic features described earlier. We incorporate both covalent and noncovalent bonds with the Euclidean distances between the atoms. For this graph neural network, we use two network architectures: 1) a variant of spatial-graph convolutional neural network (SGCNN) used in the potential net (Feinberg et al., 2018) and the fusion methods (Jones et al., 2021), and 2) a type of equivariant graph network architectures, E(n) equivariant graph neural networks (EGNN) (Satorras et al., 2021). Note that although we incorporate SGCNN and EGNN into our MIL framework, any graph neural network architectures can be utilized as a backbone model. We utilize multiple graph neural networks, each of which interprets a single instance containing a docking pose with the protein receptor. We incorporate multiple graph networks into an attention network to capture the docking poses close to the target label.

Given a bag of multiple docking pose instances in a low-dimensional embedding, we propose to use attention-network pooling to predict its single binding affinity. Although docking pose instances are generally ordered by docking scores (with the lowest energy pose listed first), the “best” docking poses (closest to the crystal structures or with the highest correlation to experimental binding affinities) are not necessarily the first ones in the set, according to the pose classification studies (Shim et al., 2022; 2024). To address this and to enable permutation-invariant MIL pooling, we use an attention-based weighted average pooling approach, similar to Luong et al. (2015), Ilse et al. (2018). In this model, the weights for each pose instance are determined by the proposed attention network, allowing for order-independent predictions.

**FIGURE 1:**
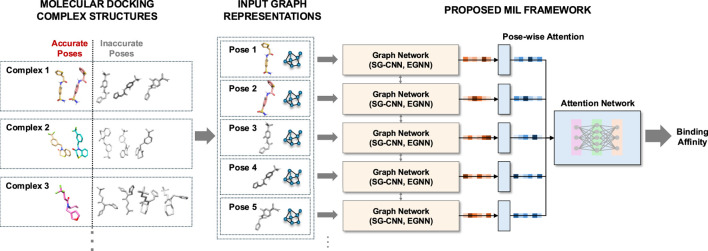
Overview of the proposed approach.

The attention network consists of an attention layer, followed by three fully-connected layers. The attention layer serves as the MIL pooling mechanism: $z=\sum_{k=1}^{K}a_{k}h_{k}$ where $a_{k}=\sigma \left(\tanh\left(Wh_{k}^{\text{T}}\right)\right)$ . It uses weight parameters (matrix) W , which are learned during training, and applies the hyperbolic tangent function tanh(⋅) element-wise. Then it applies the softmax function $\sigma \left(z\right)=\frac{e^{z_{i}}}{\sum_{j=1}^{K}e^{z_{j}}}$ to ensure that attention scores are interpretable and well-normalized. Note that we also experimented with a multi-head attention-based mechanism (Vaswani et al., 2017). However, no obvious change in prediction accuracy was observed. Figure 1 illustrates our proposed approach.

## 3 Experiment setup

In this section, we describe the experimental setup to demonstrate the effectiveness of our proposed approach. We first detail the datasets and the process for generating molecular docking poses. We provide comprehensive details on the training and evaluation procedures, together with the metrics for quantitative assessment.

### 3.1 Data

The PDBbind database (Su et al., 2019) is the most widely used dataset in structure-based machine learning studies. It combines experimental binding affinity data ( $-\log K_{i}$ or $-\log K_{d}$ ) with curated co-crystal complex structures, originally derived from the Protein Data Bank (PDB) (Burley et al., 2019). Among several versions of the PDBbind database, we use the PDBbind 2020 release, which comprises 19,443 protein-ligand co-crystal structures. The PDBbind database is divided into three subsets: *general*, *refined*, and *core*. The general set is the largest collection of the database with a broad range of complexes. The refined set comprises co-crystal complexes of better quality, curated based on resolution, binding data, and the nature of the complexes. The core set, referred to as CASF-2016, represents a smaller group of high-quality complexes meant to be representative of different protein families and serves as a benchmark for validating docking scoring and machine learning prediction methods. While the general and refined sets are used to train our proposed models, the CASF-2016 (core set) is used to evaluate our methods.

**FIGURE 2:**
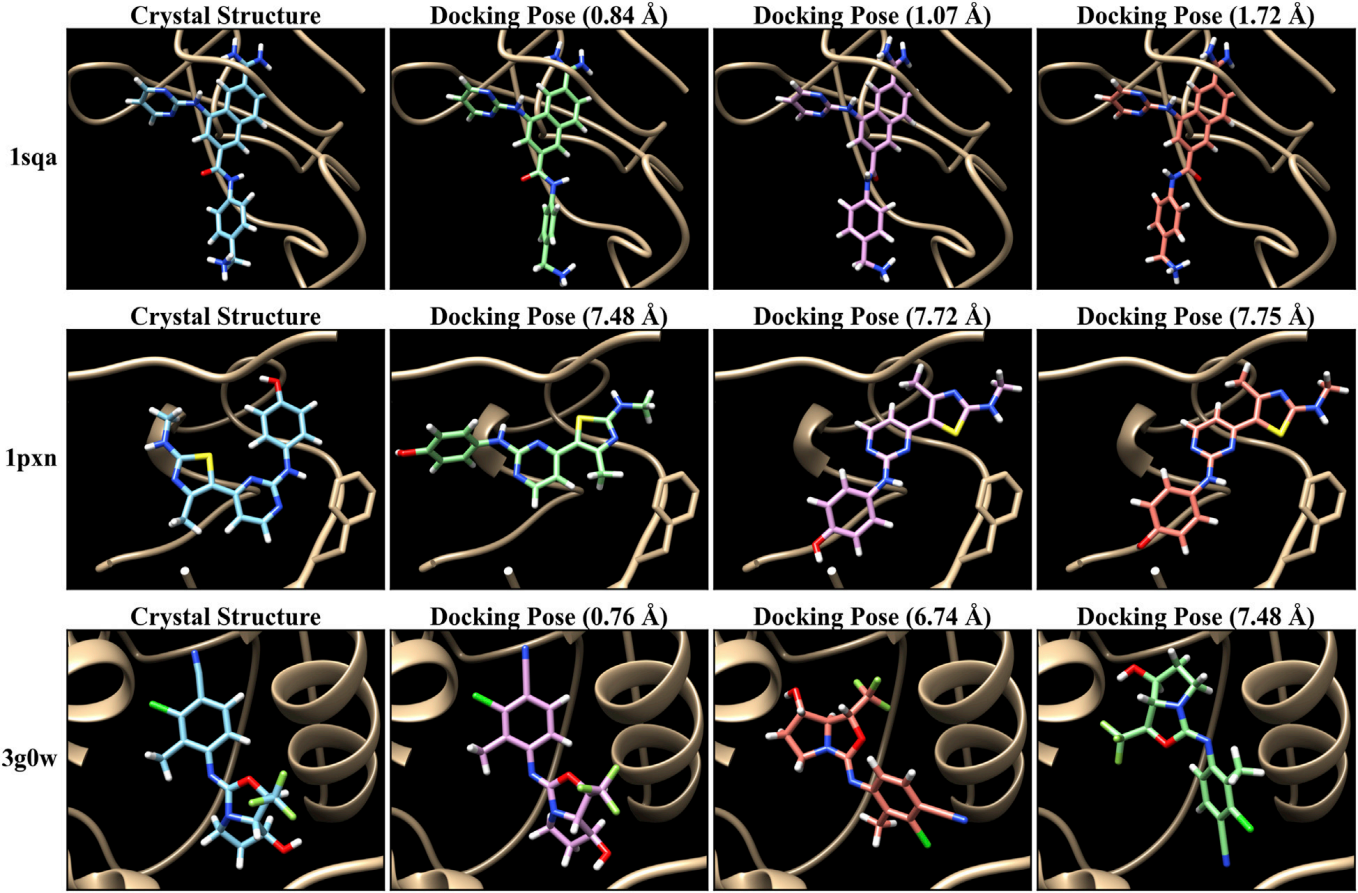
Examples of crystal structures and their docking poses of PDBbind CASF-2016 (core set). Autodock VINA (Trott and Olson, 2010; Eberhardt et al., 2021) with ConveyorLC (Zhang et al., 2014) was used for molecular docking. (⋅) indicates the RMSD error between the crystal structure and the docking pose.

**FIGURE 3:**
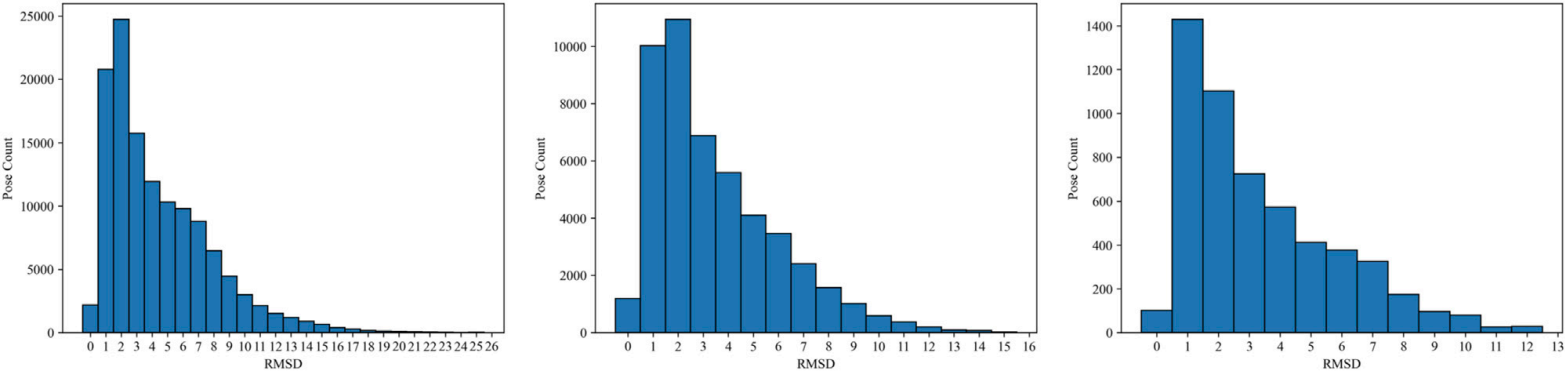
Statistics of the docking poses for training and evaluation. The plots represent the RMSD histograms between the docking poses and original crystal ligands in PDBbind 2020 general (left), refined (middle), and CASF-2016 (right) sets. Each histogram bin represents the number of poses within the specific RMSD range.

The PDBbind database provides co-crystal complex structures. Thus, we generate molecular docking poses for each of these crystal structures to demonstrate our MIL-based approach. We employed AutoDock VINA (Trott and Olson, 2010; Eberhardt et al., 2021) to generate docking poses by utilizing the ligand and receptor data with the corresponding binding site. Particularly, we used ConveyorLC (Zhang et al., 2014), a VINA docking pipeline for high-performance computing (HPC) clusters. We generated up to 10 poses for each docking ligand. We also computed the root mean square deviation (RMSD) of atomic positions between each docking pose and its corresponding crystal ligand structure using Open Drug Discovery Toolkit (ODDT) (Wójcikowski et al., 2015). Note that AutoDock VINA computes two RMSD values (lower and upper bounds) relative to the first pose, which is the top-ranked pose with the lowest score, rather than using the crystal structure as a reference. Therefore, we used the ODDT software to independently calculate RMSD values based on the crystal structures. The RMSD values serve as metrics for assessing prediction performance across various structural conformations and poses. Figure 2 shows several examples of docking poses and their RMSDs. In docking poses for the PDBbind dataset, there are cases where all poses are close to the crystal structure (poses with RMSDs below 2 Å), as shown in the top example (“1sqa”) of Figure 2. In contrast, sometimes all poses are inaccurate (poses with RMSDs exceeding 4 Å) (“1pxn”). Another case is that the majority of poses are inaccurate whereas a few pose instances are accurate (“3g0w”). Figure 3 shows the overall RMSD errors of the docking poses in the PDBbind dataset.

To evaluate our method on cases where co-crystal structures were not available, we applied our method to an additional compound dataset with molecular docking poses. We selected compound collections targeting the SARS-CoV-2 main protease receptor (“Mpro”) curated by Shim et al. (2024), aimed at identifying potential antiviral agents against the virus. The ligand SMILES data with experimental binding affinities were originally sourced from POSTERA (Morris et al., 2021) and GOSTAR (GoStar, 2023). The receptor structure was obtained from the protein data bank (pdbid: 6LU7) (Burley et al., 2019). This dataset does not provide complex crystal structures. Instead, molecular docking poses were generated using ConveyorLC (Zhang et al., 2014) for AutoDock VINA (Trott and Olson, 2010; Eberhardt et al., 2021). For each compound, we generated up to 10 docking poses. For more detail about the docking process of the Mpro dataset, refer to the original paper (Shim et al., 2024).

We used two different splitting methods to divide the entire Mpro compound set into training and test data: random split and scaffold-based split. The scaffold split ensures that similar compound structures are not present in both the training and test sets by grouping the same Bemis-Murckold scaffolds into either the training or testing set. We utilized the scaffold-based method in the ATOM Modeling PipeLine (AMPL) (Minnich et al., 2020) to generate the training and test split. The number of training and test compounds is 2,135, 1,281, respectively.

### 3.2 Training and evaluation details

We trained and evaluated our proposed models using PyTorch and PyTorch Geometric. Leveraging their distributed data parallel features, we utilized a cluster of 4 NVIDIA Titan Xp GPUs, each of which has 12 GB of memory. There are two options to train our MIL models: 1) simultaneous training: train both the backbone graph neural network and the MIL attention network together in a unified process, 2) separate training: first, train the backbone models independently and freeze the model parameters to extract features (low-dimensional embedding for 3D complexed pose structures). These features are then used to train the MIL attention network separately. For simultaneous training, we used a mini-batch size of 1 due to GPU memory constraints. While this approach can offer slightly improved predictions for some data, it is computationally expensive because it processes multiple docking pose instances (around 10 poses) with the backbone graph networks simultaneously. In contrast, the separate training option is more computationally efficient. By training the MIL attention network independently, we significantly reduced GPU resource usage and achieved more efficient computation. We used option 2 (separate training) for our experiments.

We used the RMSprop optimizer with the multi-step learning rate scheduler. The total number of epochs is 100-200, depending on the convergence of the loss function. The mini-batch size is 20. Starting with an initial learning rate of 0.001, it decreases by a factor of 0.9 at epoch intervals of 5, 10, 20, 30, 50, 70, and 90. We chose not to utilize checkpoint selection based on the validation split. Instead, we simply opted for the latest checkpoint available for our evaluation.

The hyperparameters for the SGCNN model are as follows: the thresholds for covalent and non-covalent neighbors are set to 1.5 and 4.5 Å, respectively. The size of the gated graph sequence for both covalent and non-covalent bonds is 2. The “gather” widths for covalent and non-covalent bonds are 16 and 12, respectively. We used the following hyperparameters for the EGNN model: the number of edge features is 1, and the model has 6 layers. The distance cut-off is set to 5 Å. Residual connections are used together with attention layers.

## 4 Results

We performed experiments to demonstrate the effectiveness of the proposed MIL approach using the molecular docking pose structures. Our evaluation report on the performance of our proposed approach with other existing methods used two datasets with their Autodock VINA docking poses. We present the following quantitative metrics: root mean square error (RMSE), mean absolute error (MAE), coefficient of determination $\left(r^{2}\right)$ , Pearson correlation coefficient (r) , and Spearman rank correlation coefficient (ρ) .

We benchmarked our results with those from other recent 3D structure-based methods: 1) SGCNN, a variant of Potential Net (Feinberg et al., 2018; Jones et al., 2021), 2) EGNN, equivariant graph neural network, and 3) HACNET (Kyro et al., 2023), which incorporates hybrid attentions through 3DCNN and GCN. We chose SGCNN and EGNN for comparative analysis because our method integrates the architecture into the MIL framework as backbone models. This choice allows us to analyze the behavior of our proposed MIL mechanism. Additionally, we utilized the model checkpoint for HACNET trained on crystal structures in the PDBbind 2016 general and refined sets, due to unavailability of model re-training using the PDBbind 2020 dataset. These comparative models predict binding affinities based on individual docking pose instances. One could report the quantitative metrics using all individual pose results for the comparative models. However, this method does not align with our MIL approach, which generates a single binding affinity from multiple docking poses for each complex entity. Instead, we use the following two methods. First, we report the metrics based on the predicted binding affinities for the top poses, specifically the first pose with the lowest docking scores (“Top”). This approach is commonly used in high-throughput screening with molecular docking tools. Second, we group the results based on compound IDs (e.g., PDB IDs in the PDBbind dataset), and use the average of predicted binding affinities for the same complex entity to ensure consistency with our results (“Avg”).

### 4.1 PDBbind

Table 1 summarizes the model performance on docking poses with the crystal structures in the PDBbind CASF-2016 (core set). Each complex entity can have up to 11 pose instances, including the crystal ligand instance with up to 10 docking poses. The first group (row 1–6) in Table 1 presents results from models trained exclusively on crystal structures. The SGCNN and EGNN models (row 3–6) were trained using crystal structures from the PDBbind 2020 general and refined sets. In contrast, we utilized the HACNET model (row 1–2) trained on crystal structures from the PDBbind 2016 general and refined sets. The second and third groups (row 7-9 and 10-12, respectively) show results from models trained on both crystal structures and docking poses from the PDBbind 2020 general and refined sets. “Top” presents results based on the top poses, while “Avg” shows results based on non-weighted averages across multiple docking poses within the same complex entities. The SGCNN-MIL and EGNN-MIL results present our proposed MIL method integrating the spatial graph network (SGCNN) and the equivariant graph network (EGNN) as backbone models, respectively.

**TABLE 1:** Model Performance of Binding Affinity Prediction on docking and crystal structures of the PDBbind CASF-2016. The models in the first group (row 1–6) were trained solely on the crystal structures of the PDBbind general and refined sets. HACNET was trained using the PDBbind 2016 dataset, while the other models were trained on the PDBbind 2020 dataset. The models in the second and third groups (row 7-9, and row 10–12) were trained on both docking and crystal structures of the PDBbind general and refined sets.

Method	RMSE ↓	MAE ↓	$r^{2}\;\;↑$	Pearson r↑	Spearman ρ↑
HACNET-Top (Kyro et al., 2023) (Crystal trained)	1.539	1.209	0.506	0.745	0.734
HACNET-Avg (Kyro et al., 2023) (Crystal trained)	1.523	1.202	0.512	0.763	0.750
SGCNN-Top (Feinberg et al., 2018) (Crystal trained)	1.498	1.230	0.532	0.775	0.776
SGCNN-Avg (Feinberg et al., 2018) (Crystal trained)	1.507	1.238	0.522	0.776	0.777
EGNN-Top (Satorras et al., 2021) (Crystal trained)	1.269	0.968	0.664	0.818	0.803
EGNN-Avg (Satorras et al., 2021) (Crystal trained)	1.198	0.939	0.698	0.842	0.827
SGCNN-Top	1.341	1.058	0.625	0.812	0.806
SGCNN-Avg	1.327	1.052	0.629	0.824	0.816
SGCNN-MIL (Ours)	**1.237**	**0.951**	**0.678**	**0.836**	**0.828**
EGNN-Top	1.024	0.790	0.781	0.895	0.891
EGNN-Avg	0.999	0.764	0.790	0.901	0.897
EGNN-MIL (Ours)	**0.955**	**0.736**	**0.808**	**0.904**	**0.899**

We conducted an additional experiment to show the model performance based training solely on the docking poses without the crystal ligand structures from the PDBbind CASF-2016 (core set). Table 2 shows the model performance among several methods. Similar to the results in Table 1, the first 7 rows present results from HACNET, SGCNN, EGNN, and late-fusion models, respectively, trained exclusively on crystal structures. The HACNET and fusion models (row 1,2 and 7) were trained on crystal structures from the PDBbind 2016 general and refined sets. In contrast, the SGCNN and EGNN models (row 3,4,5 and 6) were trained on crystal structures from the PDBbind 2020 general and refined sets. The second and third groups (row 8-10 and 11-13, respectively) show results from models trained exclusively on docking poses from the PDBbind 2020 general and refined sets. For each of the two backbone models (SGCNN and EGNN), we report the top-pose only (“Top”), averaged (“Avg”) across the poses, and our (“MIL”) results.

**TABLE 2:** Model Performance of Binding Affinity Prediction exclusively on docking structures of the PDBbind CASF-2016. The training sets used for the first group follow the same setup as in the previous experiment (Table 1). However, models in the second and third groups were trained solely on docking poses, without crystal structures.

Method	RMSE ↓	MAE ↓	$r^{2}\;\;↑$	Pearson r↑	Spearman ρ↑
HACNET-Top (Kyro et al., 2023) (Crystal trained)	1.539	1.209	0.506	0.745	0.734
HACNET-Avg (Kyro et al., 2023) (Crystal trained)	1.543	1.213	0.503	0.757	0.742
SGCNN-Top (Feinberg et al., 2018) (Crystal trained)	1.498	1.230	0.532	0.775	0.776
SGCNN-Avg (Feinberg et al., 2018) (Crystal trained)	1.501	1.233	0.530	0.777	0.778
EGNN-Top (Satorras et al., 2021) (Crystal trained)	1.269	0.968	0.664	0.818	0.803
EGNN-Avg (Satorras et al., 2021) (Crystal trained)	1.242	0.967	0.678	0.829	0.812
Fusion (Jones et al., 2021) (Crystal trained)	1.871	1.498	-	0.712	0.693
SGCNN-Top	1.410	1.120	0.585	0.790	0.793
SGCNN-Avg	1.423	1.130	0.578	0.788	0.788
SGCNN-MIL (Ours)	**1.242**	**0.962**	**0.678**	**0.844**	**0.838**
EGNN-Top	1.057	0.849	0.767	0.876	0.873
EGNN-Avg	1.028	0.817	0.779	0.884	0.882
EGNN-MIL (Ours)	**0.967**	**0.748**	**0.805**	**0.906**	**0.903**

Results in Tables 1, 2 show that our proposed method (“MIL”) outperforms the other methods in both cases. Overall, models trained on both crystal structures and docking poses, or solely on docking poses, outperform those trained exclusively on crystal structures. Among the SGCNN-based models (second group), our MIL method significantly outperforms the other approaches. The results from the third group (EGNN) also demonstrate that our method outperforms the other approaches.

### 4.2 SARS-CoV-2 Mpro

We conducted experiments using the SARS-CoV-2 (COVID-19) main protease dataset (“Mpro”), sourced from POSTERA (Morris et al., 2021) and GOSTAR (GoStar, 2023), and curated and docking processed by Shim et al. (2024). Our evaluations include both random splits and scaffold splits of the “Mpro” dataset. Tables 3, 4 summarize the model performance in predicting binding affinities on the dataset with random and scaffold splits, respectively.

**TABLE 3:** Model Performance of Binding Affinity Prediction on SARS-CoV-2 “Mpro” dataset with a random split.

Method	RMSE ↓	MAE ↓	$r^{2}\;\;↑$	Pearson r↑	Spearman ρ↑
SGCNN-Top	0.820	0.598	0.508	0.731	0.731
SGCNN-Avg	0.806	0.586	0.525	0.742	0.739
SGCNN-MIL (Ours)	**0.781**	**0.572**	**0.554**	**0.746**	**0.739**
EGNN-Top	0.845	0.570	0.478	0.755	0.751
EGNN-Avg	0.816	0.553	0.513	**0.770**	**0.761**
EGNN-MIL (Ours)	**0.763**	**0.535**	**0.574**	0.766	0.760

**TABLE 4:** Model Performance of Binding Affinity Prediction on SARS-CoV-2 “Mpro” dataset with a scaffold split.

Method	RMSE ↓	MAE ↓	$r^{2}\;\;↑$	Pearson r↑	Spearman ρ↑
SGCNN-Top	1.026	0.787	0.069	0.488	0.403
SGCNN-Avg	1.006	0.772	0.105	0.499	0.409
SGCNN-MIL (Ours)	**0.966**	**0.739**	**0.174**	**0.523**	**0.430**
EGNN-Top	0.929	0.689	0.236	0.602	0.502
EGNN-Avg	0.877	0.650	0.319	0.635	0.543
EGNN-MIL (Ours)	**0.846**	**0.640**	**0.366**	**0.639**	**0.550**

The evaluation results with the random split, as shown in Table 3, indicate that our proposed method generally outperforms the approaches using only the top docking pose (“Top”) or the averaged pose (“Avg”) results. However, it does not achieve the same level of performance as observed in the PDBbind evaluations. In the scaffold split scenario, where no similar compounds overlap between the training and evaluation sets, our approach clearly outperforms the other methods.

## 5 Ablation study and discussion

Our experiments with the PDBbind and Mpro datasets demonstrate that our proposed MIL approach consistently outperforms existing methods. We discuss several key observations from these experiments. First, for each backbone architecture, our MIL approach produces more accurate predictions than the non-weighted averages of the predicted binding affinities from multiple docking pose instances and the predictions from the top-ranked poses. The results demonstrate that the attention network module of our approach effectively predicts accurate binding affinities across multiple pose instances within each complex entity. In comparing the EGNN-MIL models trained on both the crystal and docking pose data with those trained on docking pose data alone, there is no significant discrepancy, as illustrated in Tables 1, 2). The model trained on both crystal and docking data exhibits slightly improved RMSE, MAE, and $R^{2}$ values, while the model trained solely on docking data shows marginal improvements in Pearson and Spearman correlations.

Second, incorporating docking poses generally improve the model prediction over training models solely on crystal structure data. For instance, SGCNN-Top and SGCNN-Avg (rows 7-8 vs. row 3–4) and EGNN-Top and EGNN-Avg (rows 10-11 vs. row 5–6) in Table 2). demonstrate this clearly. It can be argued that incorporating docking data introduces greater diversity into the data distribution, enhancing the model’s predictive performance and generalizability. In addition, adding crystal structures into the training data yields a slight improvement in model accuracy, as shown in a comparison of the results in Table 1 with those in Table 2. However, results may vary based on the quality of the docking poses for each complex structure entity. If all docking poses are significantly inaccurate relative to the crystal structures, incorporating crystal structures could enhance model accuracy.

Furthermore, the choice of backbone graph network architectures also affects the prediction accuracy. The EGNN models demonstrate a clear advantage over the SGCNN models, likely due to its effective feature learning capabilities, which enhance understanding of the input geometry of ligand-protein structures. This results in improved generalizability across different configurations (poses) within the same underlying co-complex entity.

The results evaluating the SARS-CoV-2 Mpro dataset indicate that our MIL approach achieves improved accuracy, compared to the other methods. However, the difference between the correlation coefficients (Pearson and Spearman) becomes marginal in the random split scenario. In contrast, our approach clearly outperforms other methods in the scaffold split scenario. In comparison to the results for the PDBbind dataset, the models trained on the Mpro dataset yield relatively poor performance. It is possibly due to greater inaccuracies in the Mpro docking poses compared to those in the PDBbind dataset.

### 5.1 Performance with good poses

**FIGURE 4:**
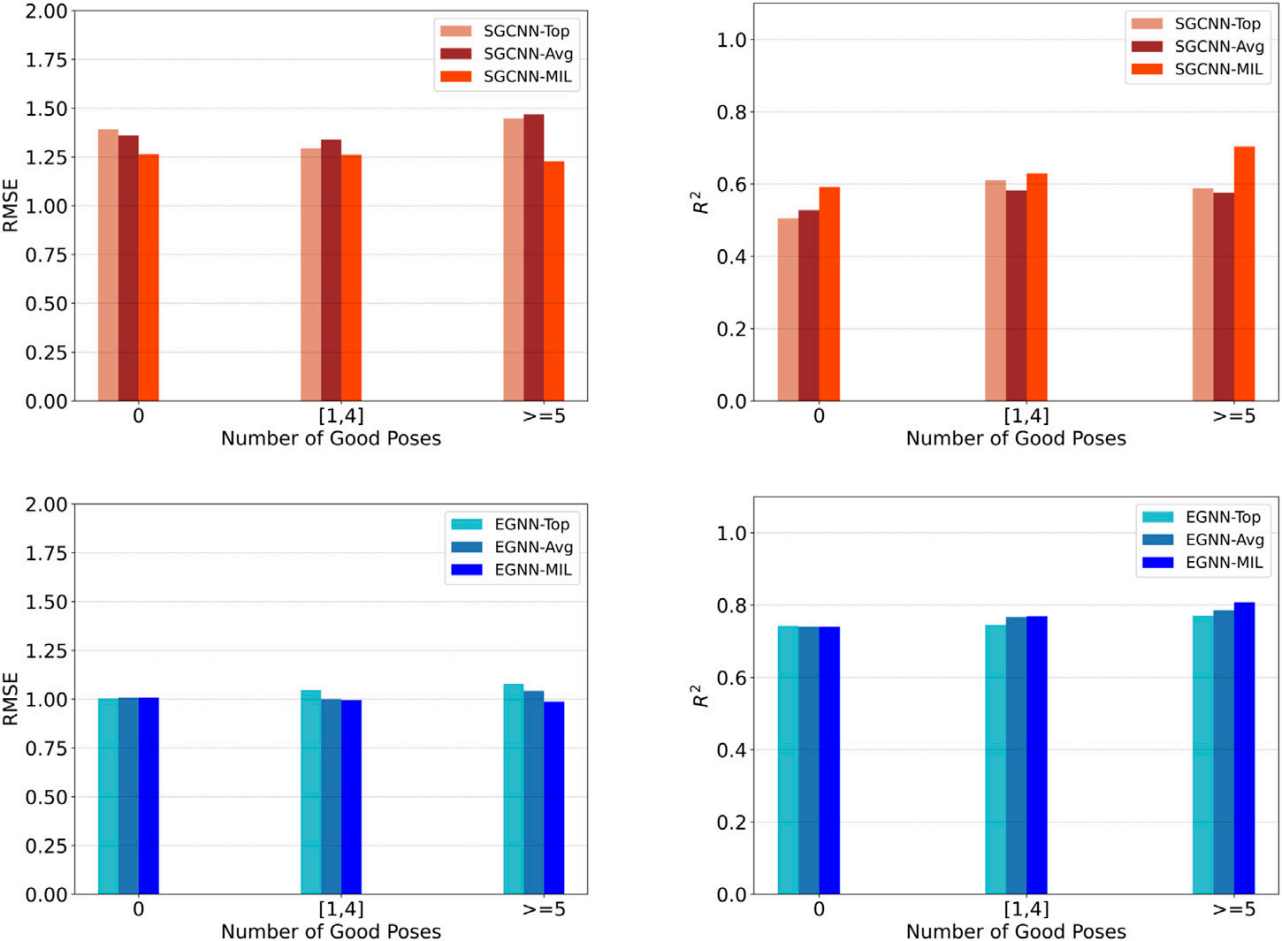
Model performance of binding affinity prediction on the PDBbind CASF-2016 dataset, based on the number of good docking poses within each complex entity. The evaluation data were divided into three groups: structures with no good poses, those with 1–4 good poses, and those with more than 4 good poses. The top and bottom row show results using the SGCNN and EGNN backbones, respectively. The left and right columns show RMSE and $R^{2}$ results, respectively.

We evaluate model performance based on the number of good docking poses within each complex entity. We define “good” docking poses as those with an RMSD less than 2.5 Å. We categorize the evaluation data into three groups: structures with no good poses, those with 1–4 good poses, and those with more than 4 good poses. Figure 4 shows model performance based on the number of good poses in the PDBbind CASF-2016 set. Compared to models that rely on top poses (Top) or average predictions across multiple poses (Avg), our approach consistently demonstrates improved and consistent performance. Notably, groups with a sufficient number of good poses tend to outperform those lacking good poses.

**FIGURE 5:**
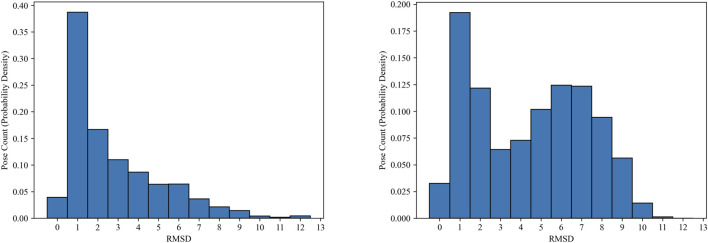
Histograms (probability density) of pair-wise RMSD errors between ligand docking poses in the PDBbind CASF-2016 (left) and SARS-CoV-2 Main Protease (right) datasets.

As we discussed earlier, models trained on the Mpro dataset generally underperform, compared to those trained on the PDBbind dataset. One possible explanation is that the quality of the docking poses (the number of “good” poses for each complex entity) may be lower in the Mpro dataset than in the PDBbind dataset. However, analyzing the quality of the docking poses in the Mpro dataset is non-trivial, due to the absence of co-crystal structures. Instead, we measure the RMSD errors between pairs of docking poses within each complex entity. Figure 5 shows the histograms of RMSD errors for pairwise ligand poses in both datasets. While these histograms do not reflect accurate poses based on crystal structures, they indicate the consistency of the docking poses for each complex. The docking poses in the Mpro dataset noticeably show greater discrepancies compared to those in the PDBbind CASF-2016 set. In real compound screening, compounds with highly inconsistent docking poses may be excluded from further ML predictions. Moreover, utilizing more advanced, state-of-the-art molecular docking simulations could improve overall prediction accuracy together with our MIL approach.

### 5.2 Optimal number of docking poses

**FIGURE 6:**
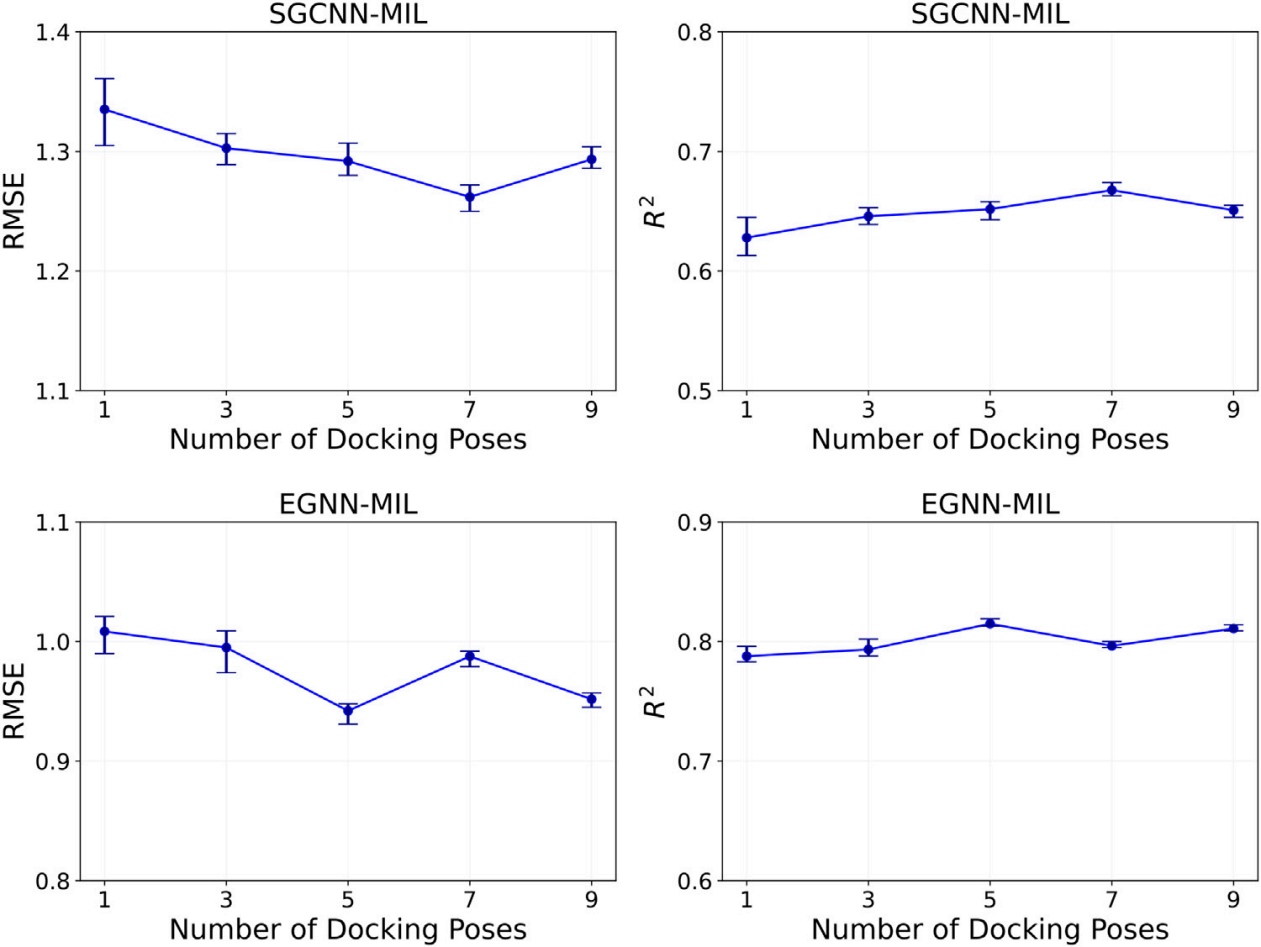
Model performance of binding affinity prediction on the PDBbind CASF-2016 dataset, based on the number of docking pose instances within each complex entity. The evaluation was performed where the number of poses is 1, 3, 5, 7, and 9. The top and bottom row show results using the SGCNN and EGNN backbones, respectively. The left and right columns show RMSE and $R^{2}$ results, respectively. Error bars indicate minimum and maximum values.

In multi-instance learning, finding an optimal range of the bag size K (the number of instances within a bag) is crucial. Larger bag sizes generally yield better feature extraction and improved model accuracy by capturing diverse instance characteristics. In contrast, larger bag sizes can increase the number of model parameters and overall complexity, which may lead to overfitting. Having more positive and negative instances can also introduce label ambiguity during model training. In our MIL setup, we generated up to 10 docking poses in most cases. We evaluated our models using varying numbers of docking pose instances for each complex entry, specifically K = 1, 3, 5, 7, and 9 instances. During model evaluation, we randomly selected K instances from 10 poses and averaged the predictions over 10 trials. Figure 6 illustrates the RMSE and $R^{2}$ values of our MIL models with two different backbone architectures. Overall, utilizing more docking poses tends to increase model accuracy. Specifically, SGCNN-MIL model achieves the best performance with 7 docking poses, while EGNN-MIL model performs optimally with 5 and 9 docking poses. While determining an optimal bag size is challenging due to factors such as compound type, the number of good poses, and model architectures, a range of 5–10 poses per bag seems to be more effective, compared to smaller bag sizes.

### 5.3 Attention types

We evaluated two different attention types for multi-instance learning. The first method involves global attention for weighted average pooling, as detailed in Section 2. The second method utilizes a multi-head attention mechanism (Vaswani et al., 2017). We observed no significant change or improvement using the multi-head attention network. While the multi-head attention mechanism offers slight improvements in predictions for SGCNN-MIL models (RMSE, MAE and $R^{2}$ ), it yields poorer results for EGNN-MIL models, as illustrated in Table 5.

**TABLE 5:** Model Performance in Binding Affinity Prediction on PDBbind CASF-2016 using two types of attention mechanisms.

Method	RMSE ↓	MAE ↓	$r^{2}\;\;↑$	Pearson r↑	Spearman ρ↑
SGCNN-MIL (Ours)	1.242	0.962	0.678	**0.844**	**0.838**
SGCNN-MIL (Multi-head)	**1.226**	**0.929**	**0.686**	0.836	0.830
EGNN-MIL (Ours)	**0.967**	**0.748**	**0.805**	**0.906**	**0.903**
EGNN-MIL (Multi-head)	1.073	0.827	0.76	0.891	0.882

### 5.4 Computational Costs

The attention network in our MIL framework is lightweight, comprising approximately 68,000 parameters, which is under 1 MB in size. The training and evaluation of our models are fast. Using a single NVIDIA Titan Xp GPU, the training time for one epoch with a batch size of 20, across 18,539 complex entities with up to 10 docking poses, is approximately 36.5 s (1.97 milliseconds per complex entity).

## 6 Conclusion

We presented a new structure-based multi-instance learning approach utilizing molecular docking poses as a bag of complex entity. Our method employs an attention network together with graph neural networks to enable permutation-invariant weighted average pooling between docking poses. We demonstrated our method using the PDBbind and the Mpro datasets. Our method offers binding affinity prediction without requiring co-crystal structures, which increases its applicability for various targets of interest.

## Data Availability

The original contributions presented in the study are included in the article/supplementary material, further inquiries can be directed to the corresponding author.
